# Servitization capabilities and network-level performance in manufacturing: evidence from the plant engineering industry

**DOI:** 10.1080/21693277.2026.2727808

**Published:** 2026-09-04

**Authors:** Maxim Ruff, Manuel Woschank

**Affiliations:** a University of Latvia, Riga, Latvia; b Chair of Industrial Logistics, Montanuniversität Leoben, Leoben, Austria

**Keywords:** Servitization capabilities, dynamic capabilities, manufacturing performance, supply chain resilience, structural equation modelling

## Abstract

In volatile manufacturing environments, particularly in project-based plant engineering, servitization is regarded as a strategic approach to enhance competitiveness and resilience. However, empirical evidence on the relationship between servitization capabilities and firm- and/or network-level performance remains limited. This study develops and tests a multi-level framework to examine how classical and digital servitization capabilities, enabled by predictive/analytical artificial intelligence (AI), link to operational performance. A sequential mixed-methods design combined expert interviews and structural equation modelling based on survey data. Qualitative findings identified digitalization, customer relationship management, and operational excellence as key capability domains. Quantitative results show that neither classical nor digital servitization capabilities significantly link to firm-level performance. Instead, both capabilities strongly link to network-level coordination, collective operational performance, and supply chain resilience. The findings shift the performance focus of servitization from individual firms to manufacturing ecosystems and highlight network orchestration and capability renewal as key drivers of competitive advantage.

## 1. Introduction

Manufacturing firms, particularly in the capital-intensive plant engineering sector, face unprecedented competitive pressures. Global supply chains remain vulnerable to geopolitical tensions, raw-material volatility, and climate-related disruptions, while customers increasingly demand customised, outcome-based solutions rather than isolated products. At the same time, digital transformation and stringent sustainability requirements force manufacturers to rethink traditional product-centric business models. In this environment, servitization as the strategic integration of advanced services with physical products has emerged as a critical response. By shifting from one-time sales to long-term value co-creation, manufacturers can stabilise revenue streams, deepen customer relationships, and build operational resilience across complex production ecosystems (Baines et al., 2009a; Kowalkowski et al., 2017).

The industrial services market illustrates the momentum behind this shift. Valued at USD 39.1 billion in 2023, it is projected to reach USD 59.1 billion by 2028, growing at a compound annual rate of 8.6% (MarketsandMarkets, 2024). Drivers include the proliferation of Industry 4.0 technologies such as IoT, digital twins, predictive maintenance, and artificial intelligence (AI), which enable the seamless integration of products and services and increasingly act as capability enablers rather than standalone performance drivers (Camarella et al., 2024). Classic examples illustrate the potential: Rolls-Royce’s ‘Power-by-the-Hour’ model transformed aircraft engine sales into guaranteed availability contracts; Hilti moved from selling power tools to fleet-management-as-a-service; and Alstom’s Train-Life services ensure continuous operational readiness for rail operators (Davies, 2007; Johnson et al., 2008; Smith, 2013). These cases highlight how servitization creates hybrid offerings that deliver superior customer value while generating recurring revenue and counter-cyclical cash flows (Wise & Baumgartner, 1999).

The focus of this study is the bulk-solids sector of the international plant engineering industry, a B2B context characterised by large capital-good projects, long asset lifecycles, high engineering specificity, and intensive collaboration with EPC contractors (e.g, CTCI, Tecnimont, or Samsung E&A). This industry has received limited empirical attention in servitization research despite its structural distinctiveness. EPC contractors occupy a pivotal intermediate role between equipment manufacturers and end customers, shaping value delivery through specification authority, procurement processes, and project coordination. The study draws on survey data from 154 international plant engineering professionals, predominantly from German-speaking Europe and internationally active firms in the bulk-solids sector. This contextual focus matters because the industry is a globally significant, export-oriented segment in which servitization and network coordination are strategically consequential.

Academic and practitioner literature has long emphasised the strategic benefits of servitization. It fosters competitive differentiation, builds entry barriers, and develops dynamic capabilities that enhance adaptability in volatile markets (Baines et al., 2009a; Seclen-Luna et al., 2025; Teece, 2007). Yet empirical reality is far more nuanced. The well-documented ‘service paradox’ shows that many manufacturers invest heavily in services only to see margins erode or overall performance stagnate (Gebauer et al., 2005). Studies report mixed, sometimes contradictory results: positive linear relationships in some contexts (Raddats et al., 2019) versus negative or insignificant relationships in others (Benedettini et al., 2017; Neely, 2008), as well as U-shaped relationships and profitability hurdles depending on context, intensity, and timing (Fang et al., 2008; Homburg et al., 2022; Visnjic & van Looy, 2012). These contradictions persist partly because existing research rarely distinguishes clearly between firm-level and network-level performance outcomes, and partly because it concentrates on general manufacturing or B2C contexts while paying limited attention to the specific conditions of project-based, engineering-intensive industries.

This study addresses these gaps by examining how classical and digital servitization capabilities influence operational performance in manufacturing firms. Two research questions guide the investigation:

RQ1:
**How are classical and digital servitization capabilities associated with firm-level and network-level operational performance in the plant engineering industry?**
RQ2:
**How is organisational artificial intelligence adoption associated with servitization capabilities and operational performance, and how are these associations reflected through dynamic capabilities, Service-Dominant Logic, and contractor strategic innovation?**

The central contribution is both theoretical and practical. Theoretically, the paper shifts the performance locus of servitization from the isolated firm to the manufacturing ecosystem, demonstrating that value creation in complex, project-based environments occurs primarily at the network level. It resolves apparent contradictions in the literature by separating firm-level from network-level outcomes, refutes the prevailing assumption that servitization directly boosts firm-level performance metrics, and clarifies the enabling, rather than direct performance, role of AI. Practically, the findings offer operations and production managers in plant engineering concrete guidance: rather than expecting immediate firm-level ROI, they should prioritise network orchestration, ecosystem governance, and capability renewal.

The remainder of the paper is organised as follows. Section 2 develops the theoretical framework and derives the hypotheses. Section 3 describes the sequential mixed-methods design and PLS-SEM methodology. Section 4 presents qualitative and quantitative empirical results. Section 5 discusses theoretical contributions and managerial implications. Section 6 concludes with limitations and avenues for future research.

## 2. Theoretical framework and hypotheses

Manufacturing firms in capital-intensive industries such as plant engineering operate under intense pressure from volatile supply chains, rising sustainability demands, and accelerating digitalisation. In this context, servitization represents a promising pathway to stabilise revenue, deepen customer relationships, and strengthen operational resilience. However, the performance implications of servitization remain contingent and often disappointing, a phenomenon known as the service paradox (Gebauer et al., 2005). To explain when and how servitization capabilities translate into superior outcomes, this study adopts a focused three-theory lens that integrates the Resource-Based View (RBV), the Dynamic Capabilities View (DCV, placed centrally), and Service-Dominant Logic (SDL, applied selectively where value co-creation is empirically relevant at the network level). To avoid duplication, hypothesis development is integrated directly into the presentation of each theory rather than separated into a distinct section.

### 2.1. Resource-based view

The Resource-Based View (Barney, 1991; Wernerfelt, 1984) provides the foundational logic: sustained competitive advantage stems from valuable, rare, inimitable, and non-substitutable (VRIN) resources. In plant engineering, these include specialised engineering know-how, installed-base data, and established customer relationships, assets that are particularly difficult to replicate in project-based environments. The RBV explains why some manufacturers achieve superior servitization outcomes while others do not: performance heterogeneity arises from differences in resource endowments and the ability to bundle them into hybrid service offerings.

### 2.2. Dynamic capabilities view

Building directly on the RBV, the Dynamic Capabilities View constitutes the central theoretical pillar (Teece et al., 1997; Teece, 2018). Dynamic capabilities are defined as the firm’s ability to sense opportunities and threats, seize them through resource mobilisation, and transform the resource base to maintain alignment with changing environments. In servitization contexts, sensing identifies shifting customer demands for outcome-based solutions; seizing involves reallocating engineering and service resources to develop hybrid offerings; and transforming requires organisational reconfiguration (e.g. new KPIs, cross-functional teams, digital platforms) to deliver services at scale. Empirical servitization research consistently shows that dynamic capabilities explain performance variance more strongly than static resource positions alone (Coreynen et al., 2017; Kohtamäki et al., 2020). For manufacturing firms, the DCV bridges the gap between possessing resources and deploying them successfully under volatility, precisely the condition required for operational performance and supply chain resilience.

Digital capabilities, as a subset of dynamic capabilities, are particularly technology-intensive and require longer maturation periods in traditional plant engineering ecosystems. According to Teece (2018) and Coreynen et al. (2017), the integration of digital technologies into established industrial routines demands organisational learning, cultural adaptation, and accumulated experiential knowledge before the reconfiguration logic of dynamic capabilities can fully unfold. This theoretical reasoningnot post-hoc empirical observation, grounds the expectation, developed below, that mediation relationships are initially more pronounced for classical than for digital servitization capabilities.

### 2.3. Service-dominant logic (selective application)

Service-Dominant Logic (Vargo & Lusch, 2004, 2016) complements the framework selectively, where value co-creation becomes empirically salient. SDL reframes goods as distribution mechanisms for service provision and posits that value is always co-created through resource integration by multiple actors, including the beneficiary. In plant engineering ecosystems, this perspective is particularly relevant for network-level outcomes: manufacturers, EPC contractors, and end customers jointly integrate resources (data, expertise, infrastructure) to realise value-in-use.

Importantly, SDL is not positioned here as a co-equal theory alongside the RBV and the DCV. Rather, it serves a specific empirical function: it explains the relational and ecosystem dimension of servitization performance, particularly the observed divergence between firm-level and network-level link. Empirically, SDL is operationalised as a mediating construct (five items measuring resource integration and value co-creation practices) whose indirect relationship on network-level performance is tested alongside dynamic capabilities. This constrained role reflects the selective-application principle and avoids the theoretical over-burdening that can arise when SDL is invoked foundationally but not implemented empirically.

### 2.4. Integrated framework and hypothesis development

**Figure 1.**
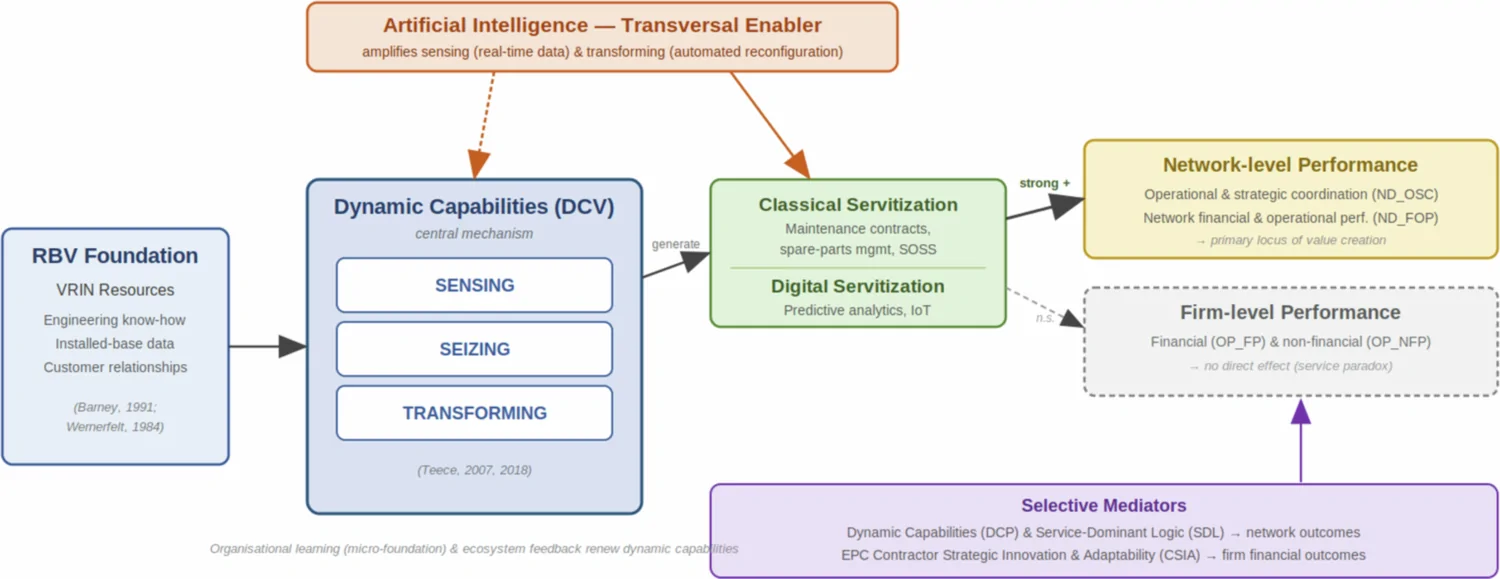
Integrated framework. RBV resources feed central DCV processes, which generate first-order servitization capabilities; AI is a transversal enabler; performance is realised primarily at the network level, with selective mediation via DCP, SDL, and CSIA.

The three perspectives are synthesised into a single, coherent framework: ‘Servitization Capabilities as Dynamic Capabilities in Manufacturing Ecosystems’. This framework posits that servitization capabilities are not isolated routines but manifestations of higher-order dynamic capabilities operating within multi-actor manufacturing ecosystems. RBV resources form the necessary foundation; DCV processes (sensing, seizing, transforming) provide the mechanisms that generate first-order classical and digital servitization capabilities; AI acts as a transversal enabler that amplifies the sensing and transforming processes; and SDL specifies how value is co-created and captured at the network level. The mediators (provider dynamic capabilities, SDL, and EPC contractor strategic innovation and adaptability) are positioned on the paths linking capabilities to the two distinct performance levels. Figure 1 illustrates the integrated framework.

On this basis, six focused hypotheses are derived. Each follows directly from the theory presented above.

H1:
**Classical servitization capabilities of the provider are positively associated with network-level operational and strategic coordination as well as network financial and operational performance in plant engineering ecosystems.**

Classical servitization capabilities (e.g. strategic and organisational support for service offerings, maintenance contracts, spare-parts management) represent the ability to seize and transform existing VRIN resources into hybrid offerings (Baines et al., 2009a; Teece, 2018). In project-based manufacturing ecosystems dominated by EPC contractors, such capabilities primarily materialise at the network level through improved coordination routines and collective performance. Where industry-specific evidence is scarce, this expectation is supported by findings from adjacent project-based and B2B servitization contexts (Coreynen et al., 2017; Kohtamäki et al., 2020); the application to bulk-solids plant engineering is novel.

H2:
**Digital servitization capabilities of the provider are positively associated with network-level operational and strategic coordination as well as network financial and operational performance in plant engineering ecosystems.**

Digital servitization capabilities (e.g. predictive analytics, remote monitoring, IoT-enabled services) extend classical capabilities through technology-enabled resource reconfiguration (Coreynen et al., 2017; Kohtamäki et al., 2020). In plant engineering, where assets have long lifecycles and high downtime costs, these capabilities allow real-time data integration across the ecosystem. SDL underscores that value-in-use is co-created through digital resource integration among manufacturers, EPC contractors, and end customers.

H3:
**Artificial intelligence integration is positively associated with both classical and digital servitization capabilities of the provider.**

AI functions as a transversal enabler that amplifies sensing (real-time data analytics), seizing (predictive decision support), and transforming (automated reconfiguration) processes (Manser Payne et al., 2021; Teece, 2018). From an RBV perspective, AI upgrades existing engineering resources into higher-order capabilities. Consistent with recent empirical findings, AI is positioned as a capability enabler rather than a direct performance driver (Grubic, 2018).

H4:
**The positive relationship between classical servitization capabilities and network-level performance is associated with the provider's dynamic capabilities.**

Dynamic capabilities act as the central mechanism that converts first-order servitization capabilities into network-level outcomes (Teece, 2007). In complex plant engineering projects, sensing market and customer needs, seizing service opportunities, and transforming routines across organisational boundaries require higher-order orchestration that is particularly pronounced at the network level.

H5:
**The positive relationship between classical servitization capabilities and firm-level financial performance is associated with contractor strategic innovation and adaptability.**

EPC contractors are pivotal orchestrators in plant engineering ecosystems. Their strategic innovation and adaptability enable manufacturers to translate servitization capabilities into tangible firm-level financial returns through appropriate project margins and risk sharing. This mediation path is grounded in SDL (value co-creation through partner integration) and the Open-System perspective (Katz & Kahn, 1978). The focus on classical rather than digital capabilities in H4 and H5 follows directly from the DCV maturation logic set out in Section 2.2: digital capabilities, being more novel and technology-intensive, require longer learning cycles before they can function as reliable antecedents of mediated performance relationships (Coreynen et al., 2017; Teece, 2018), whereas classical services, rooted in established resource bundles, provide a more immediate basis for value transformation through mediation mechanisms. This is an ex-ante theoretical expectation, not a post-hoc rationalisation.

H6:
**(Exploratory): Classical and digital servitization capabilities are not expected to exhibit significant positive associations with firm-level operational or financial performance.**

This proposition captures the theoretically and empirically critical service paradox in manufacturing (Gebauer et al., 2005). While servitization capabilities create substantial value at the network level, they do not automatically translate into direct firm-level performance gains due to high initial investment costs, organisational resistance, and the need for ecosystem-level coordination (Benedettini et al., 2017; Visnjic & van Looy, 2012). H6 is framed as an exploratory proposition regarding a boundary condition of the model rather than a standard directional hypothesis; it is stated here for conceptual completeness, while its empirical status is interpreted alongside the results (Section 4.4) and discussed in relation to the service paradox (Section 5).

**Figure 2.**
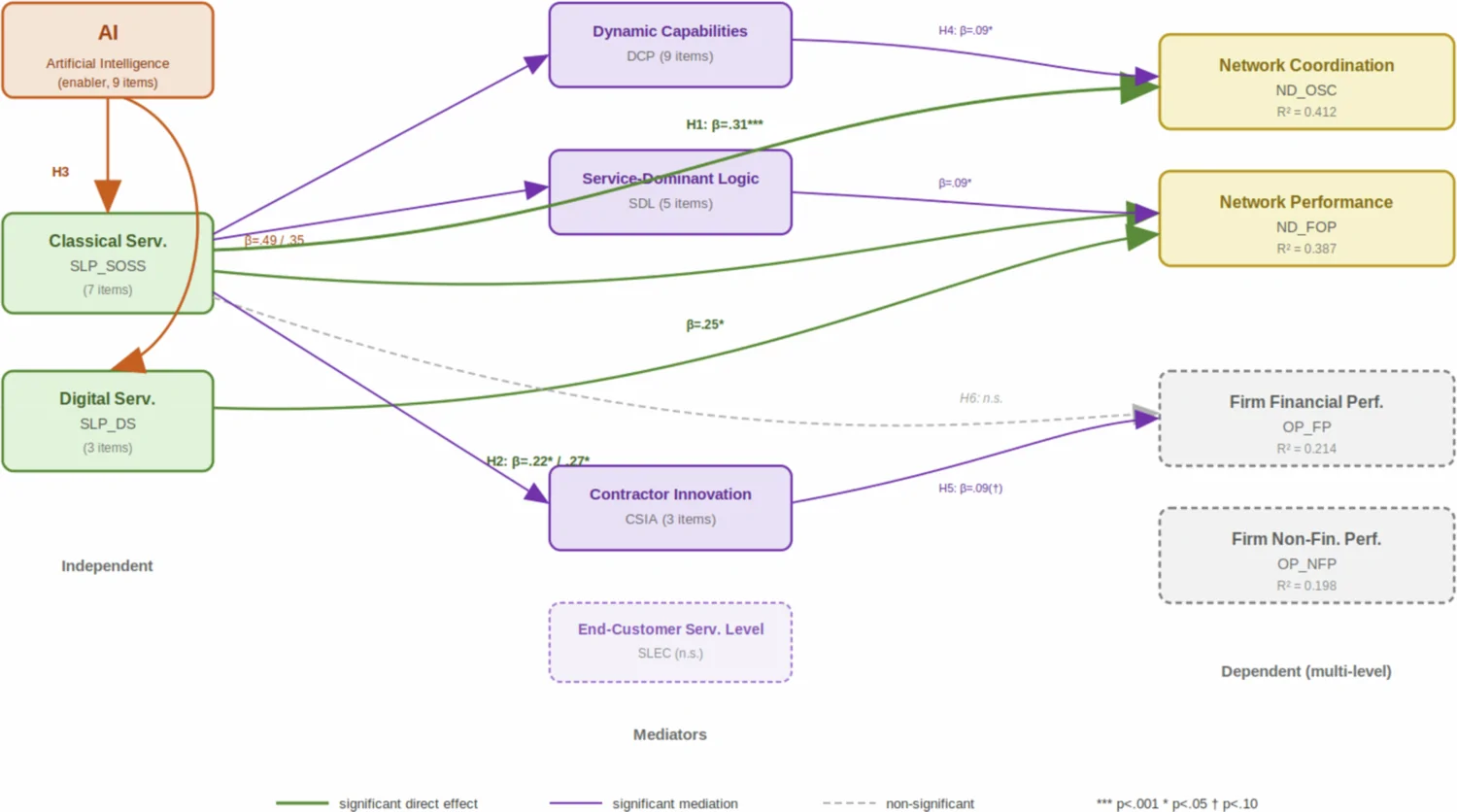
Research model with hypothesised relationships. Servitization capabilities are expected to drive network-level performance directly; firm-level relationships are expected to be non-significant (service paradox); AI enables capabilities; mediation runs selectively through classical capabilities.

Figure 2 presents the resulting research model with its hypothesised paths.

## 3. Methodology

This study employs a sequential, exploratory, mixed-methods design combining qualitative depth with quantitative breadth (Creswell & Plano Clark, 2017). The rationale for this design follows the sequential triangulation logic: the qualitative phase provides contextual grounding and construct refinement, while the quantitative phase subjects these insights to statistical testing across a broader population, thereby strengthening validity through methodological complementarity (Bell et al., 2022; Greene et al., 1989; Morse & Niehaus, 2009). Sequential triangulation is particularly appropriate here because it allows the richness of practitioner perspectives, captured in expert interviews, to inform measurement instruments and model specification before large-scale testing, counteracting the abstraction bias common in purely deductive survey research. The unit of analysis is the firm (the manufacturing provider): respondents served as key informants reporting on firm-level capabilities and on the provider’s network relationships, and network-level constructs are therefore measured as the focal firm’s perception of its network (Jack & Raturi, 2006).

### 3.1. Qualitative phase

Seventeen semi-structured expert interviews were conducted between August and October 2022 (average duration of 63 minutes). Participants included general managers, vice presidents of service and business development, heads of lifecycle solutions, and senior service executives from international plant engineering firms and EPC contractors. One additional interview was conducted with a leading academic expert in industrial services. Participants were selected via purposive sampling based on three criteria: (1) a senior hierarchical position with strategic or operational responsibility for servitization, (2) at least ten years of domain experience, and (3) active involvement in servitization initiatives. Fourteen participants were recruited through the researcher’s professional network; three via snowball sampling. Interviews were conducted face-to-face (*n* = 4) or via videoconference (*n* = 13), audio-recorded with informed consent, and transcribed verbatim (total corpus: 267 pages). Transcripts were systematically anonymised. Before each interview, participants were informed of the study’s purpose, the voluntary nature of their participation, and the intended use of the data for academic research; verbal informed consent was obtained from all participants before audio recording commenced.

Data analysis was performed in MAXQDA 24 following Kuckartz, (2014, Kuckartz & Rädiker, 2024) structured qualitative content analysis (QCA), which combines deductive and inductive coding. This method was preferred over Mayring’s predominantly deductive approach because the evolving nature of servitization research requires openness to emergent empirical patterns alongside theory-guided coding (Kuckartz, 2014). An initial category system was derived from the theoretical framework (deductive) and then iteratively refined through constant comparison as new themes emerged from the material (inductive). Empirical subcategories were grouped into conceptual categories reflecting the theoretical constructs, which were in turn aggregated into higher-order dimensions distinguishing internal capabilities from external conditions (Corbin & Strauss, 2015; Gioia et al., 2013). MAXQDA’s Code Matrix Browser and Code Relations Browser were used to explore co-occurrence patterns and relationships between categories, enhancing transparency and traceability.

Coding transparency and traceability were addressed as follows. All coding was conducted by the lead researcher; given the single-coder design, formal intercoder reliability statistics were not computed. To mitigate the limitations of single-coder analysis, the codebook was developed iteratively against the theoretical framework, coding decisions and category definitions were documented systematically in the MAXQDA project file to ensure an auditable trail, and category boundaries were refined through repeated passes over the material with constant comparison. Each excerpt reported below is linked to its interview ID and position marker (e.g. ID 4, Pos. 2–3) in the MAXQDA project file, and the complete transcripts and coding output are available upon request for academic review, subject to data protection requirements. The reliance on a single coder is acknowledged as a limitation in Section 6.

### 3.2. Quantitative phase: sample and data collection

The target population comprised mid- to senior-level managers with strategic or operational responsibility for servitization initiatives in the international plant engineering sector. Purposive sampling ensured relevance and expertise; we note that, by design, this is non-probability sampling, with implications for generalisation discussed in Section 6.

Data were collected via an online survey (LimeSurvey Cloud) between 26 May and 30 June 2024. The questionnaire was distributed through professional networks, industry associations, and direct contacts. Of 246 responses received, 172 were fully completed. After rigorous data cleaning (response-time analysis, Mahalanobis distance for multivariate outliers, and systematic pattern detection), 154 usable questionnaires remained for analysis.

The final sample is highly experienced: 73.4% of respondents had more than ten years of professional experience, and 44.2% worked in sales or service-related functions. Firm sizes were well distributed across large, medium, and smaller plant engineering companies. The study was conducted primarily in the German-speaking region of Europe and with internationally active firms in the bulk-solids plant engineering sector; this context is explicitly acknowledged as a boundary condition for generalisation.

Non-response bias was assessed by comparing early (first 30%) and late (last 30%) respondents on all key constructs (Armstrong & Overton, 1977). No significant differences emerged (t-tests, *p* > .05). Representativeness for the plant engineering population is supported by the alignment of the firm-size distribution and functional-role profile with industry characteristics; however, the absence of a complete industry sampling frame limits a formal representativeness claim. Sample adequacy was assessed via the inverse square root method (Kock & Hadaya, 2018), yielding a minimum recommended *n* of 155 for the smallest expected path coefficient; our *N* = 154 nearly meets this threshold, with the method’s slight overestimation tendency providing additional assurance.

### 3.3. Measurement instruments

All constructs were measured reflectively using multi-item Likert-type scales (1 = strongly disagree to 7 = strongly agree). Items were adapted from established sources and refined through the qualitative phase, a pilot study (*n* = 44), and a pretest (*n* = 16) to ensure content validity and contextual fit for plant engineering. The complete questionnaire, including all item wordings and source attributions, is provided in Appendix A. Table 1 presents the final measurement items with their sources.

**Table 1.** Measurement items (Final Scales with Source Attributions).

Construct (Code)	No.	Representative Items	Sources
Strategic & Organisational Support of Servitization (SLP_SOSS): classical capabilities	7	Our leaders promote future offerings that include servitization. We review our progress in the servitization transformation with top management.	Gebauer et al. (2012); Paschou et al. (2020)
Digital Servitization (SLP_DS): digital capabilities	3	Our organisation uses smart technology to improve our service offering. We use data analytics to create value for our customers.	Kohtamäki et al. (2020); Bustinza et al. (2017)
Artificial Intelligence (AI): enabler	9	AI helps us predict service maintenance needs before they become critical. AI-driven analytics significantly influence our strategic decisions regarding service offerings.	Manser Payne et al. (2021); adapted from Grubic (2018)
Network Operational & Strategic Coordination (ND_OSC): network DV	7	Our network can respond quickly to changes in global market conditions. Coordination between network units to optimise overall performance is highly effective.	Adapted from Kohtamäki et al. (2020); Teece (2007)
Network Financial & Operational Performance (ND_FOP): network DV	2	We use targeted performance metrics to monitor the financial and operational performance of our network.	Developed for this study
Organisational Financial Performance (OP_FP): firm DV	4	Our sales amount is very high. Our return on investment is very high.	Adapted from Gebauer et al. (2012)
Organisational Non-Financial Performance (OP_NFP): firm DV	4	Our company is able to grasp the right timing for launching new products or services. We vigorously invest in the development of new technology.	Adapted from Kohtamäki et al. (2020)
Contractor Strategic Innovation & Adaptability (CSIA): mediator	3	EPC contractors proactively adapt their strategies and processes to changing market conditions.	Developed for this study (plant engineering context)
Servitization Level of the End Customer (SLEC): mediator	4	Our end customers recognise the innovation in our solutions.	Adapted from Paschou et al. (2020)
Dynamic Capabilities of the Provider (DCP): mediator	9	We are effective in transforming existing information into new knowledge. We can successfully reconfigure our resources to develop new productive assets.	Teece (2007, 2018)
Service-Dominant Logic (SDL): mediator	5	Our organisation integrates resources from various partners to enhance our service offerings. Our organisation encourages customer input throughout the value creation process.	Vargo & Lusch (2004, 2016)

Construct-to-model mapping: SLP_SOSS captures classical servitization capabilities and SLP_DS captures digital servitization capabilities (independent variables); AI is an independent enabling construct; CSIA, SLEC, DCP, and SDL function as mediators; ND_OSC and ND_FOP are network-level dependent variables; and OP_FP and OP_NFP are firm-level dependent variables. SLEC was retained in the measurement model for completeness as a theorised mediator, but no significant indirect relationship via SLEC were found in the structural model (all *p* > .10).

### 3.4. Data analysis procedure

Data were analysed using SmartPLS 4 (version 4.1.0.6). PLS-SEM was chosen for its suitability with complex models incorporating both reflective constructs and predictive objectives in a relatively novel research domain (Hair et al., 2017, 2022). All constructs were modelled as reflective.

The measurement model was evaluated for internal consistency (Cronbach’s *α* and CR > 0.70), convergent validity (AVE > 0.50), and discriminant validity (HTMT < 0.85; Fornell–Larcker criterion). The structural model was evaluated for collinearity (VIF < 3.0), explanatory power (R^2^), predictive relevance (Q^2^ via blindfolding, omission distance = 7), and path significance (bootstrapping, 5,000 subsamples, bias-corrected accelerated confidence intervals). Effect sizes (f^2^) were interpreted per Cohen’s guidelines. Common method bias was assessed via Harman’s single-factor test and full collinearity VIF (Kock, 2015); no single factor exceeded 50% of variance, and all VIF values remained below 3.3.

## 4. Results

### 4.1. Qualitative findings: core capability dimensions

The qualitative analysis of 17 expert interviews (267 pages of transcribed data) yielded ten primary categories comprising 45 subcategories, providing a systematic framework of the competencies required for successful servitization in plant engineering. Detailed information of the interview participants can be found in Appendix B. Moreover, Table 2 presents the category system.

**Table 2.** Main categories and subcategories from qualitative content analysis.

Category	Key Subcategories	Role in Model
1. Servitization Strategy & Business Model Transformation	Business model transformation; shift to servitization; transactional vs. servitization models; value proposition realignment	Informs SLP_SOSS construct
2. Digitalisation & Data Utilisation	Digital platforms; data analytics; IoT integration; data management	Informs SLP_DS and AI constructs
3. CRM & Market Understanding	Customer value perception; long-term relationships; understanding customer needs; trust	Informs SLEC and CSIA constructs
4. Organisational Capabilities & Transformation	Competence development; leadership & cultural change; cross-functional collaboration; change management	Informs DCP construct
5. Operational Excellence & Technological Innovation	Operational efficiency; predictive maintenance; process optimisation; technological integration	Informs SLP_SOSS and SLP_DS
6. Market & Competitive Environment	Economic influences; market adaptation; competitive strategies	External condition (not modelled)
7. Strategic Partnerships & Ecosystems	EPC contractor collaboration; building business ecosystems; strategic partnerships	Informs CSIA and ND_OSC constructs
8. Financial & Market Considerations	Revenue streams; investment & risk management; financial strategies	External outcome dimension
9. Sustainability & Environmental Impact	Environmental regulations; sustainable business practices	External condition (not modelled)
10. Employee Engagement & Talent Management	Talent acquisition; employee training; competency development	Embedded in DCP (micro-foundation)

Categories 1–5 and 10 represent genuine organisational servitization capabilities (internally embedded and manageable); Categories 6, 8, and 9 reflect external conditions; Category 7 occupies an intermediate position as an orchestration capability. This theoretical distinction directly informed the quantitative model specification. Three categories emerged as particularly salient across interviews, digitalisation and data utilisation, customer relationship management, and operational excellence, and were prioritised in construct development. The qualitative phase also validated the distinction between classical and digital servitization, confirming that practitioners experience them as conceptually and operationally distinct, though interdependent.

Selected interview excerpts illustrate the key findings (all excerpts anonymised; interview IDs and position markers refer to the MAXQDA project file).

**On the strategic and organisational dimension of servitization (SLP_SOSS):**

“When are we actually integrating the service perspective into product development, so that, from a service standpoint, we can already say: this could be built in now, which would give us a unique selling point for servitization later?” (ID 7, Pos. 3)

**On digital servitization capabilities (SLP_DS):**

“In every tender we receive, it is now required that we use specific software platforms, so that we can collaborate within the digital environment of our customers.” (ID 4, Pos. 2)

**On AI as capability enabler:**

“Market intelligence, where we are currently investing in software tools: how can we bring together our millions of data points on delivered equipment (installed base) with market data? That is what I am trying to marry up, to derive: when does which gearbox come in for overhaul?” (ID 3, Pos. 2)

**On network-level coordination and EPC contractor orchestration (ND_OSC/CSIA):**

“We are building libraries of all our components and products, which we share across our own network at all locations, and which we subsequently hand over to contractors during project execution. Orchestrating this network is a capability we need.” (ID 4, Pos. 2–3)

“The contractor’s business model creates a bottleneck: they want as many comparable offers as possible, to commission the cheapest. That contradicts our approach of generating added value. So, we now engage with investors much earlier, before the EPC is even selected.” (ID 4, Pos. 3)

**On the service paradox and the absence of direct firm-level performance relationships (H6):**

“There are scientific studies that say: everybody wants to go digital, companies buy servers, expensive equipment, but performance does not grow in the same step. It is usually a U-shape: you invest, but first you have to build it up, and only then does performance, the cash flow, possibly arrive.” (ID 5, Pos. 2)

**On ecosystem-level value creation:**

“The challenge is not the innovation itself. The much bigger challenge is breaking up the established decision-making processes, and you can only do that if you truly understand what the customer needs and how value translates across the entire value chain.” (ID 14, Pos. 2)

These excerpts illustrate how qualitative evidence mapped onto the quantitative constructs and hypotheses. The transition from qualitative to quantitative followed the sequential-triangulation logic: qualitative themes informed construct operationalisation, item wording was validated against interview language, and the MAXQDA category system guided hypothesis specification (Section 2.4).

### 4.2. Descriptive statistics

Descriptive statistics for all constructs (7-point Likert scale) are shown in Table 3. Means ranged from mid- to upper levels (3.5–5.0), indicating generally positive evaluations of servitization-related practices. The highest means were observed for organisational non-financial performance (OP_NFP, M = 5.02) and the servitization level of the end customer (SLEC, M = 4.99), while network operational and strategic coordination (ND_OSC) showed the lowest (M = 3.52), suggesting that network-level coordination remains a developmental challenge in the industry.

**Table 3.** Descriptive statistics (*N* = 154).

Construct	N	Mean	SD	Skewness	Kurtosis
AI	154	3.997	1.251	−0.298	−0.621
CSIA	154	3.909	0.973	−0.054	0.124
DCP	154	4.377	0.906	−0.060	−0.955
ND_OSC	154	3.520	1.097	0.150	−0.406
ND_FOP	154	4.705	1.146	−0.418	−0.017
OP_FP	154	4.919	1.019	−0.563	0.654
OP_NFP	154	5.016	1.116	−0.880	1.305
SDL	154	4.651	0.989	−0.275	0.394
SLEC	154	4.992	0.842	−0.258	−0.038
SLP_SOSS	154	3.802	1.170	0.097	−0.040
SLP_DS	154	4.000	1.247	−0.364	−0.093

### 4.3. Measurement model evaluation

All constructs were modelled as reflective. Table 4 presents the reliability and validity statistics.

**Table 4.** Reliability and validity statistics.

Variable	No. of Items	Cronbach’s α	CR	AVE	IIC
SLP_SOSS	7	0.914	0.928	0.684	0.603
SLP_DS	3	0.849	0.910	0.771	0.656
AI	9	0.956	0.965	0.755	0.694
ND_OSC	7	0.884	0.902	0.649	0.524
ND_FOP	2	0.798	0.908	0.831	0.664
OP_FP	4	0.858	0.905	0.705	0.858
OP_NFP	4	0.789	0.913	0.841	0.484
CSIA	3	0.789	0.876	0.703	0.556
SLEC	4	0.806	0.872	0.630	0.510
DCP	9	0.930	0.927	0.863	0.599
SDL	5	0.873	0.909	0.666	0.583

All Cronbach’s *α* and CR values exceed 0.70. AVE values are above 0.50, confirming convergent validity. Discriminant validity is established by HTMT ratios below 0.85 (all upper 95% CI bounds below the threshold) and adherence to the Fornell–Larcker criterion. Indicator loadings ranged from 0.712 to 0.934 (all *p* < 0.001). Common method bias was ruled out (Harman’s single-factor test < 50% variance; full collinearity VIF < 3.3). The measurement model is robust and meets all recommended thresholds (Hair et al., 2022).

### 4.4. Structural model results

The structural model demonstrates good overall fit (SRMR = 0.072; NFI = 0.912) and no critical collinearity (VIF < 2.8). It explains substantial variance in the endogenous constructs (R^2^: ND_OSC = 0.412, ND_FOP = 0.387, OP_FP = 0.214, OP_NFP = 0.198) and demonstrates adequate predictive relevance (Q^2^ > 0 for all). Table 5 reports the selected path coefficients.

**Table 5.** Structural model: selected path coefficients.

Path	β	t-value	*p*-value	95% BCa CI	f^2^	Result
SLP_SOSS → ND_OSC	0.312	4.821	< 0.001	[0.198; 0.467]	0.142	Supported
SLP_SOSS → ND_FOP	0.248	2.112	0.035	[0.003; 0.359]	0.089	Supported
SLP_SOSS → OP_FP	0.012	0.043	0.965	[−0.223; 0.206]	0.001	Not supported
SLP_SOSS → OP_NFP	0.008	0.031	0.937	[−0.198; 0.215]	0.001	Not supported
SLP_DS → ND_OSC	0.219	2.134	0.033	[0.011; 0.270]	0.071	Supported
SLP_DS → ND_FOP	0.267	2.309	0.016	[0.052; 0.441]	0.096	Supported
SLP_DS → OP_FP	0.089	0.772	0.440	[−0.145; 0.312]	0.008	Not supported
SLP_DS → OP_NFP	0.142	1.692	0.091	[−0.023; 0.310]	0.021	Not supported
AI → SLP_SOSS	0.492	7.341	< 0.001	[0.365; 0.619]	0.318	Supported
AI → SLP_DS	0.351	5.128	< 0.001	[0.218; 0.483]	0.160	Supported

Of 32 tested indirect relationships, only three were significant: SLP_SOSS ⟶ CSIA ⟶ OP_FP (*β* = 0.094, *p* = 0.054; marginally significant), SLP_SOSS ⟶ DCP ⟶ ND_OSC (*β* = 0.089, *p* = 0.013), and SLP_SOSS ⟶ SDL ⟶ ND_FOP (*β* = 0.089, *p* = 0.022). No significant indirect relationships emerged for digital servitization or AI on any performance outcome, and AI shows no direct relationships on performance outcomes (all *p* > 0.07). Full path tables, R^2^ decomposition, and robustness cheques are available in the supplementary material.

The empirical results confirm a theoretically coherent pattern: servitization capabilities primarily enhance network-level performance, with no significant direct relationships on firm-level outcomes. This robustly confirms the service paradox in capital-intensive manufacturing (consistent with the exploratory proposition H6). The three significant indirect paths, all involving classical capabilities, highlight the contingent and mediated nature of firm-level financial returns, consistent with the DCV maturation logic developed in Section 2.2.

The effect-size pattern reinforces this interpretation. Across the model, the two AI paths display the largest f^2^ values in the model (0.318 and 0.160, respectively), exceeding Cohen, (1988) threshold for a medium-to-large effect and substantially outweighing every other path; this corroborates, at the level of practical rather than merely statistical significance, the theorised role of AI as the strongest capability enabler in the framework. By contrast, all four direct paths from servitization capabilities to firm-level performance are negligible by both criteria simultaneously (f^2^ ≤ 0.021, p ≥ 0.091), indicating that the absence of a direct effect reflects a robust null relationship rather than an artefact of limited statistical power. The modest R^2^ values for firm-level performance (OP_FP = 0.214; OP_NFP = 0.198) are therefore attributable almost entirely to the marginally significant CSIA mediation path rather than to any direct effect of servitization capabilities, consistent with the selective mediation logic developed in Section 2.4.

## 5. Discussion

The empirical results paint a clear and theoretically coherent picture: servitization capabilities in the plant engineering industry create substantial value, but almost exclusively at the network level. Both classical and digital servitization capabilities significantly link to operational and strategic coordination and collective financial and operational performance across the ecosystem. Artificial intelligence emerges as a powerful enabler that strengthens both capability types, yet neither servitization capabilities nor AI show any direct positive relationship on firm-level performance. Only three indirect paths proved significant, all involving classical servitization capabilities. These findings confirm the service paradox in a manufacturing context (Gebauer et al., 2005) and shift the performance locus from the isolated firm to the manufacturing ecosystem.

### 5.1. Theoretical contributions

This study advances servitization research in three important ways. First, it provides robust empirical evidence for the network-centric nature of value creation in project-based manufacturing, a perspective that has been conceptually discussed but rarely tested with large-scale SEM data (Gebauer et al., 2012; Kohtamäki et al., 2020). By distinguishing firm-level from network-level performance, the paper resolves apparent contradictions between studies reporting positive servitization relationships (Raddats et al., 2019) and those reporting negative or insignificant relationships (Benedettini et al., 2017; Neely, 2008). The resolution lies in ecosystem coordination: previous studies that conflate firm and network performance naturally find mixed results because the causal locus differs by level of analysis. This is a theoretically novel contribution that goes beyond merely acknowledging the service paradox to explaining its structural cause in project-based B2B industries.

Second, the study clarifies the role of AI as a transversal capability enabler rather than a direct performance driver, a nuanced positioning that reconciles optimistic practitioner accounts with more cautious empirical findings (Grubic, 2018; Manser Payne et al., 2021). The empirical pattern is consistent with AI’s infrastructural role: it amplifies the sensing and transforming processes that generate servitization capabilities, but value realisation requires additional organisational and network-level mechanisms. Notably, the AI paths also display the largest effect sizes in the entire structural model (Section 4.4), indicating that this enabling role is not merely statistically detectable but practically substantial.

Third, the study demonstrates that dynamic capabilities and SDL operate most effectively when embedded in ecosystem coordination rather than in isolated firm strategies. SDL’s empirical role, as a mediating construct on one significant indirect path (SLP_SOSS → SDL → ND_FOP), is precisely delimited: it explains value materialisation at the network level through resource integration and value co-creation logic, consistent with its selective-application principle in the theoretical framework. This restrained but theoretically precise use of SDL contributes a more disciplined integration than prior multi-theory frameworks in which SDL appears as a co-equal foundational theory but is not empirically implemented as such. We also note that our model builds on, rather than wholesale adopts, prior firm-level servitization-capability taxonomies such as the Servitization Maturity Model (Paschou et al., 2020), tailoring the capability set to the multi-level, EPC-centred plant engineering context.

Together, these contributions offer a parsimonious three-theory framework (RBV as foundation, central DCV, and selective SDL) that is both theoretically rigorous and empirically testable in manufacturing settings, a more modest and defensible theoretical architecture than frameworks that invoke SDL as a parallel foundational lens without fully implementing it in the measurement model.

### 5.2. Managerial implications

For production and operations managers in plant engineering, the central implication is clear: do not expect immediate firm-level ROI from servitization investments. Value accrues first at the network level, and firm-level financial returns materialise only through indirect mechanisms, particularly contractor strategic innovation and adaptability.

Operations and supply-chain managers should treat EPC contractors as strategic innovation partners, not mere executors. They should actively develop contractors’ strategic innovation and adaptability through joint capability-building programmes, shared innovation roadmaps, and aligned incentive systems, and should design contracts and governance mechanisms that explicitly reward joint coordination and knowledge sharing. Implementing shared performance dashboards that track network-level metrics (e.g. overall equipment effectiveness across the project chain, end-to-end response time) rather than only internal KPIs helps make ecosystem value visible and manageable.

Senior production leaders should invest in cross-company digital platforms enabling real-time data exchange for predictive maintenance, the area where AI delivers its highest leverage. Practical steps include establishing a ‘network operations council’ with key EPC partners to review servitization progress quarterly, redirecting part of the digital transformation budget from isolated AI pilots to joint ecosystem platforms, and revising performance management systems to include network-level contribution targets in incentive schemes for operations and supply-chain executives.

## 6. Conclusions

This study set out to examine how classical and digital servitization capabilities relate to operational performance and supply chain resilience in manufacturing firms, with a particular focus on the project-based plant engineering industry. Drawing on a focused theoretical framework (RBV as foundation, central DCV, and selective SDL) and a rigorous mixed-methods analysis, combining qualitative content analysis of 17 expert interviews with PLS-SEM analysis of 154 international respondents, the findings deliver a clear message: servitization creates substantial value, but almost exclusively at the network level.

Four core empirical findings stand out. First, both classical and digital servitization capabilities exert significant positive direct relationships on network-level operational and strategic coordination and on collective financial and operational performance. Second, neither capability type shows a statistically significant direct relationship on firm-level performance, a pattern that robustly confirms the service paradox in capital-intensive manufacturing. Third, AI functions as a powerful transversal enabler that significantly strengthens both servitization capability types yet does not directly translate into performance gains. Fourth, mediation is selective and meaningful: provider dynamic capabilities and SDL channel relationships to network outcomes, while contractor strategic innovation and adaptability links classical servitization capabilities to firm-level financial returns. The principal theoretical contribution is the relocation of the servitization performance locus from the isolated firm to the manufacturing ecosystem, which in turn resolves the long-standing contradictory-findings debate.

### 6.1. Limitations and future research

Several limitations should be noted. The cross-sectional design precludes causal conclusions. The sample is focused on one industry segment, bulk-solids plant engineering, primarily in the German-speaking region of Europe, and results may differ in less project-oriented or less EPC-centric manufacturing sectors; the use of purposive, non-probability sampling further constrains statistical generalisation. All measures are perceptual; objective performance data would strengthen validity. Firm-level financial performance remains only modestly explained (R^2^ = 0.214), suggesting additional unmodelled contingencies (e.g. pricing models, risk-sharing agreements). Finally, the qualitative coding was conducted by a single researcher; although coding decisions were documented systematically and refined iteratively against the theoretical framework to ensure an auditable trail, the absence of a second independent coder and of formal intercoder reliability statistics is a limitation, and future qualitative work would benefit from multiple coders and formal agreement measures.

Future research should adopt longitudinal, or panel designs to examine capability development trajectories over time. Comparative studies across industries would test the boundary conditions of the network-centric relationship. Objective performance measures and multi-informant designs (manufacturer + EPC contractor + end customer) would further enhance robustness. Experimental or intervention-based studies could test whether targeted network-governance interventions accelerate the translation of servitization capabilities into firm-level returns. Finally, future work should examine how the maturation of digital capabilities over time eventually closes the mediation gap currently observed for digital versus classical servitization.

## Data Availability

The quantitative survey data supporting the findings of this study are available from the corresponding author upon reasonable request. The qualitative interview transcripts generated during the study are not publicly available owing to the confidentiality obligations towards participants; anonymised excerpts are presented in the manuscript, and the full MAXQDA coding output is available upon request for academic review purposes, subject to data-protection requirements.

## Appendices

### Appendix A: complete questionnaire (main study)

All items use a 7-point Likert scale (1 = strongly disagree; 7 = strongly agree). Sources are indicated in brackets.

**Strategic & Organisational Support of Servitization (SLP_SOSS)**

[Adapted from Paschou et al., 2020 and Gebauer et al. (2012)]

- RM1: Our employees understand the shift to servitization.
- RM2: The organisation invests in the competencies and capabilities to deliver servitised offerings.
- RM3: Our business cases and key performance indicators are linked to our servitization roadmap.
- TMO1: Our leaders understand the importance of the servitization transformation.
- TMO2: Our leaders promote future offerings that include servitization.
- TMO3: We review our progress in the servitization transformation with top management.
- OSC1: We keep improving our internal processes to deliver our servitization offerings more efficiently.

**Digital Servitization (SLP_DS)**

[Kohtamäki et al., 2020; Bustinza et al., 2017]

- DS1: Our organisation uses smart technology to improve our service offering.
- DS2: We use data analytics to create value for our customers.
- DS3: We use digital platforms to connect our services with our customers.

**Artificial Intelligence (AI)**

[Adapted from Manser Payne et al., 2021 and Grubic (2018)]

- AI1: AI helps us predict service maintenance needs before they become critical issues.
- AI2: We use AI to analyse customer data and improve service personalisation.
- AI3: AI-driven analytics significantly influence our strategic decisions regarding service offerings.
- AI4: AI tools enhance our ability to monitor and manage our service operations in real time.
- AI5: AI enables us to identify patterns in customer behaviour that improve our service delivery.
- AI6: We use AI to automate routine service tasks, freeing up resources for complex service activities.
- AI7: AI supports our decision-making process in optimising service delivery and resource allocation.
- AI8: The integration of AI into our services has improved our response time to customer issues.
- AI9: AI-based tools are essential for developing innovative service offerings in our organisation.

**Network Operational & Strategic Coordination (ND_OSC)**

[Adapted from Kohtamäki et al., 2020; Teece (2007)]

- OSC1: Our network can respond quickly to changes in global market conditions.
- OSC2: Coordination between network units to optimise overall performance is highly effective.
- OSC3: Our network demonstrates strong adaptability in the face of unexpected challenges.
- OSC4: Information sharing across network units is timely and effective.
- OSC5: Our network has clear mechanisms for resolving conflicts among network members.
- OSC6: Strategic alignment among all network partners is consistently maintained.
- OSC7: Our network leverages collective strengths to achieve competitive advantages.

**Network Financial & Operational Performance (ND_FOP)**

[Developed for this study]

- FOP1: We use targeted performance metrics to monitor the financial and operational performance of our network.
- FOP2: Our network consistently achieves its financial performance targets.

**Organisational Financial Performance (OP_FP)**

[Adapted from Gebauer et al. (2012)]

- FP1: Our sales amount is very high.
- FP2: Our return on investment is very high.
- FP3: Our profit margin is very high.
- FP4: Our revenue growth over the past three years has been very strong.

**Organisational Non-Financial Performance (OP_NFP)**

[Adapted from Kohtamäki et al., 2020]

- NFP1: Our company is able to grasp the right timing for launching new products or services.
- NFP2: We vigorously invest in the development of new technology.
- NFP3: Our organisation responds quickly to competitive challenges.
- NFP4: We attract and retain high-quality talent effectively.

**Contractor Strategic Innovation & Adaptability (CSIA)**

[Developed for this study; plant engineering context]

- CSIA1: EPC contractors proactively adapt their strategies and processes to changing market conditions.
- CSIA2: EPC contractors consistently demonstrate innovative approaches in their project management.
- CSIA3: EPC contractors effectively integrate new technologies and methodologies into their operations.

**Servitization Level of the End Customer (SLEC)**

[Adapted from Paschou et al., 2020]

- SLEC1: Our end customers recognise the innovation in our solutions.
- SLEC2: Our end customers actively engage in co-creating value with us.
- SLEC3: Our end customers are open to outcome-based service agreements.
- SLEC4: Our end customers invest in developing long-term partnerships with us.

**Dynamic Capabilities of the Provider (DCP)**

[Teece, (2007, 2018)]

- DC1: We are effective in transforming existing information into new knowledge.
- DC2: We can successfully reconfigure our resources to develop new productive assets.
- DC3: Our organisation quickly integrates new processes and technologies in response to market changes.
- DC4: We actively scan the environment to identify new business opportunities in servitization.
- DC5: Our organisation is effective in seizing market opportunities through rapid reallocation of resources.
- DC6: We continuously learn from our service delivery experiences to improve future performance.
- DC7: Our organisation fosters innovation through continuous improvement of our service delivery processes.
- DC8: We actively manage and coordinate internal and external resources to support service innovation.
- DC9: Our organisation encourages and rewards innovative thinking among employees.

**Service-Dominant Logic (SDL)**

[Vargo & Lusch, (2004, 2016)]

- SDL1: Our organisation integrates resources from various partners to enhance our service offerings.
- SDL2: Our organisation encourages and facilitates customer input throughout the value creation process.
- SDL3: We actively co-create value with our customers through collaborative service development.
- SDL4: Our service offerings are designed to enable customers to achieve their desired outcomes.
- SDL5: We view our products as platforms for delivering ongoing service and value to our customers.

### Appendix B: interview participants

**Table B1.** Overview of expert interview participants.

Code	Position	Institution (Type/Region)	Category
ID 1	Professor of Business Administration	University of Applied Sciences (Germany)	Academic Expert
ID 2	General Manager, Business Unit	International Plant Engineering Firm (Germany)	Manufacturer
ID 3	Head of Business Development, Non-Automotive Aftermarket	Global Automotive Supplier (Germany)	Manufacturer
ID 4	General Manager, Business Unit	International Plant Engineering Firm (Germany)	Manufacturer
ID 5	Managing Director, Service Compounding and Extrusion	International Plant Engineering Firm (Asia-Pacific)	Manufacturer
ID 6	President, Division	International Plant Engineering Firm (USA)	Manufacturer
ID 7	Vice President, Business Unit	Global Industrial Group (Germany)	Manufacturer
ID 8	Head of Service Technology and Products	International Plant Engineering Firm (Germany)	Manufacturer
ID 9	Head of Service Sales	International Plant Engineering Firm (Germany)	Manufacturer
ID 10	General Manager, Business Unit	International Plant Engineering Firm (Switzerland)	Manufacturer
ID 11	General Manager, Business Unit	International Plant Engineering Firm (Germany)	Manufacturer
ID 12	General Manager, Business Unit	International Plant Engineering Firm (Germany)	Manufacturer
ID 13	Senior Manager, Life-Cycle Solutions	Global Power Systems Provider (Germany)	Manufacturer
ID 14	President, Division (Polymer)	International Plant Engineering Firm (Germany)	Manufacturer
ID 15	General Manager, Business Unit	International Plant Engineering Firm (Germany)	Manufacturer
ID 16	General Manager, Business Unit	International Plant Engineering Firm (Switzerland)	Manufacturer
ID 17	Senior Manager, Marine Service Sales & Contract Execution	Global Power Systems Provider (Germany)	Manufacturer
